# CD39 on CD8^+^ Tumour‐Infiltrating Lymphocytes: A Paradoxical Marker of Tumour Reactivity and Immunosuppression

**DOI:** 10.1002/eji.70288

**Published:** 2026-10-08

**Authors:** Manolo Sambucci, Daniela Fenoglio, Francesca Ferrera, Laura Camporesi, Andrea Lagazio, Giovanna Borsellino, Luca Battistini, Gilberto Filaci

**Affiliations:** ^1^ Department of Internal Medicine and Medical Specialities (DIMI) University of Genova Genoa Italy; ^2^ Neuroimmunology Unit LIFE Institute for Research and Health Care ‐ Santa Lucia IRCCS Rome Italy; ^3^ Biotherapy Unit, IRCCS Azienda Ospedaliera Metropolitana Genoa Italy; ^4^ Department of Experimental Medicine (DIMES) University of Genoa Genoa Italy

**Keywords:** adenosine, CD8^+^ TILs, CD39, immune checkpoint blockade, T‐cell exhaustion, tumour microenvironment, tumour reactivity

## Abstract

CD39 (ENTPD1) occupies a paradoxical position in the immunology of solid tumours. On CD8^+^ tumour‐infiltrating lymphocytes (TILs), its surface expression serves as one of the most reliable available markers of genuine tumour‐antigen experience and identifies the clonally expanded tissue‐resident and cytolytically competent T cells, that are largely absent from the bystander populations, which are specific for tumour‐unrelated antigens and numerically dominate many tumour infiltrates. Yet the same enzymatic activity that defines CD39, the hydrolysis of extracellular ATP (eATP) to AMP, directly feeds CD73‐dependent adenosine generation. These very cells therefore actively perpetuate the immunosuppressive purinergic milieu that constrains their own effector function and that of surrounding immune cells. CD39^+^CD8^+^ TILs thus represent simultaneously the best cellular evidence that a productive anti‐tumour immune response has occurred, and among the most potent local drivers of its suppression. This review places this paradox at its centre, dissecting the molecular basis of CD39 expression, its two opposing functional identities across solid tumour types and the therapeutic logic that follows: Blocking CD39 activity could simultaneously release the suppressive brake and preserve the tumour‐reactive cells that express it.

## Graphical abstract

On tumour‐reactive CD8^+^ TILs, CD39 drives two opposing fates at once: It marks cells actively cytolysing tumour cells via released ATP, yet also degrades that same ATP into adenosine, triggering A2AR signalling that locks the cells into a hypoxia‐amplified, self‐reinforcing immunosuppressive loop, blunting their own anti‐tumour action.

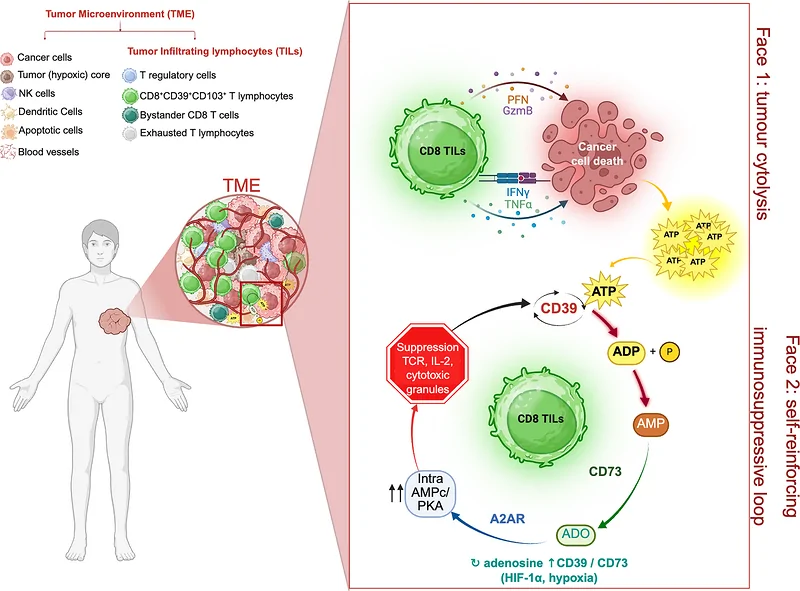

## 1 Introduction

Immune checkpoint blockade (ICB) has transformed the treatment landscape of multiple solid cancers, yet durable responses remain confined to a minority of patients, and reliable cellular biomarkers of response are still lacking. Tumour‐infiltrating CD8^+^ T cells (TILs) are central mediators of ICB efficacy, but the intratumoural CD8^+^ compartment is deeply heterogeneous: It encompasses both tumour‐antigen‐specific effectors capable of direct cytolysis and bystanders, recruited through non‐specific inflammatory signals but specific for tumour‐unrelated antigens and therefore not activated by tumour antigen within the tumour [1, 2]. This heterogeneity critically limits the predictive value of bulk TIL quantification and undermines efforts to stratify patients for immunotherapy. The challenge, therefore, is not simply to count CD8^+^ T cells but to identify which of them have actually seen the tumour.

In many solid tumours, the CD8^+^ TILs that have engaged tumour targets do not emerge from that encounter as potent effectors, and they progressively deteriorate. This process is termed T‐cell exhaustion and is characterised by sustained co‐inhibitory receptor expression (PD‐1, TIM‐3, LAG‐3 and TIGIT), loss of effector cytokine polyfunctionality, epigenetic locking of a dysfunctional transcriptional state and profound metabolic reprogramming [3, 4]. Notably, the metabolic dimension of exhaustion, which involves mitochondrial dysfunction, impaired oxidative phosphorylation and altered purinergic signalling, is increasingly recognised as a key and underappreciated determinant of T‐cell dysfunction that inhibitory receptor phenotyping alone does not capture [5, 6]. Conventional exhaustion markers, such as PD‐1, TIM‐3, and LAG‐3, are informative, high or co‐expressed levels of these receptors are enriched in tumour‐reactive clones [7], but their expression alone does not reliably discriminate exhaustion from physiological chronic activation, and none of them directly mediates an immunosuppressive effector function within the tumour microenvironment CD39 does.

The ectonucleoside triphosphate diphosphohydrolase CD39 (*ENTPD1*) has emerged as a marker that uniquely bridges antigen experience and metabolic exhaustion in CD8^+^ TILs. Its expression is induced by chronic TCR stimulation and is tightly coupled to the transcriptional exhaustion programme governed by TOX and NR4A factors, making it a reliable surrogate of sustained tumour‐antigen encounter [8, 9, 10]. CD39 particularly when co‐expressed with the tissue‐residency integrin CD103 identifies tumour‐reactive CD8^+^ TIL clones with high specificity across multiple solid malignancies, and is largely absent from bystander populations [10, 11]. So far, so useful. But CD39 enzymatic activity hydrolyses extracellular ATP (eATP) that represents a pro‐inflammatory danger signal that sustains T‐cell activation and generates immunosuppressive adenosine through a CD73‐dependent axis that directly feeds back to suppress the function of the very cells that express it [12, 13, 14]. This is the paradox at the heart of CD39 biology in cancer.

In this review, we examine the molecular biology of CD39 on CD8^+^ TILs, dissect its two faces as a marker of tumour reactivity and as a driver of immunosuppression, analyse how the paradox manifests across different tumour types and explore how understanding it reframes prognostic interpretation and therapeutic strategy.

## 2 The Molecular Biology of CD39 on CD8^+^ T Cells

CD39 (*ENTPD1*) is a plasma membrane‐anchored ectonucleotidase that hydrolyses eATP and ADP sequentially to AMP; CD73 then converts AMP to adenosine [15, 16, 17]. Adenosine signals through the A2A receptor (A2AR) to elevate intracellular cAMP, suppressing TCR signalling, IL‐2 production and cytotoxic granule release in T cells, NK cells and myeloid effectors [18, 19, 20, 21, 22]. Beyond adenosine generation, by hydrolysing eATP, CD39 does not merely generate a suppressive signal, it simultaneously eliminates the pro‐inflammatory P2X receptor costimulation that sustains T‐cell activation. CD39 expression on CD8^+^ T cells is induced by chronic TCR stimulation and co‐regulated with the TOX‐driven exhaustion programme [9, 23, 24]; hypoxia amplifies both CD39 and CD73 through HIF‐1α, directly linking tumour oxygenation to purinergic immunosuppressive tone [25, 26, 27, 28]. Single‐cell analyses across multiple solid tumours confirm that *ENTPD1* expression is concentrated in terminally exhausted CD8^+^ clusters co‐expressing *HAVCR2*, *LAG3*, *TIGIT* and *TOX*, and essentially absent from TCF‐1^+^ progenitor‐exhausted or bystander populations [29, 30, 31, 32]. This transcriptional and spatial selectivity, induced only where chronic antigen encounter has occurred, is the mechanistic foundation of the paradox this review addresses.

## 3 The Two Faces of CD39 on CD8^+^ TILs

Understanding CD39 on CD8^+^ TILs requires holding two things in mind simultaneously. The same molecule that tells us a T cell has seen the tumour is also the molecule that is actively making the environment around that T cell less immunogenic. We examine each face in turn, before asking what happens when we look at both together across different cancers.

### 3.1 Face 1: CD39 as a Marker of Tumour Reactivity

CD39 cuts through the bystander noise with remarkable specificity. Duhen et al. [10] demonstrated that CD39^+^CD8^+^ TILs accumulate selectively in tumour‐invaded tissue, and precisely primary tumours and metastatic lymph nodes, but are absent from non‐invaded nodes and peripheral blood [10]. It is important to note, this is not a feature of bystanders. Enzymatic activity was confirmed in these cells, as was an impaired ability to produce IFNγ, TNF and IL‐2, consistent with antigen‐driven exhaustion rather than naïve or bystander activation [33, 34, 35]. Of particular note, CD39 expression on tumour‐infiltrating CD8^+^ T cells associates with evidence of TCR‐based antigen recognition: clonal expansion within the tumour, oligoclonal TCR repertoires distinct from those in peripheral blood and direct MHC Class I‐restricted cytolysis of autologous tumour cells [10].

The co‐expression of CD39 with the tissue‐residency integrin CD103, which mediates retention in epithelial tissues through E‐cadherin binding, provides a particularly refined criterion to identifying for tumour‐reactive TILs. Across six solid malignancies, Duhen et al. [10] showed that CD39^+^CD103^+^ double‐positive (DP) CD8^+^ TILs were selectively enriched for tumour reactivity, whereas single‐positive and double‐negative subsets were not. The DP population exhibited the highest levels of PD‐1, TIM‐3 and CD69, hallmarks of tissue‐resident, chronically stimulated T cells, the most focussed, tumour‐specific TCR clonotypes. Similar findings have since been reported in lung cancer [36], where CD39^+^CD103^+^ TILs co‐express the highest transcriptomic scores for tumour reactivity by CITE‐seq [11, 37], in studies across melanoma cancer using single‐cell analyses [38, 39, 40] and high‐grade serous carcinoma [41].

### 3.2 Face 2: CD39 as a Driver of Immunosuppression

The same cells that carry the immunological memory of tumour encounter are, by virtue of expressing CD39, actively catabolising the eATP that would otherwise sustain their own activation. This is not a theoretical consequence; it is a demonstrable functional reality. Canale et al. [8] showed that CD39^+^CD8^+^ TILs suppressed the IFNγ production of co‐cultured responder T cells in *trans*, a contact‐ and soluble‐factor‐mediated bystander suppression activity previously considered a Treg‐specific function [42, 43, 44, 45].

The mechanism is biochemically direct. High concentrations of eATP released from dying tumour cells and metabolically stressed lymphocytes create a pro‐inflammatory substrate that, under normal circumstances, would amplify local immunity through P2RX7 and P2RX4 signalling on T cells [15]. CD39 on exhausted TILs drains this reservoir, converting it into AMP; the terminal step to adenosine, however, depends on CD73, which at least in humans is largely absent from tumour‐reactive CD39^+^ T cells themselves and is instead expressed by tumour cells and other TME populations, so that adenosine generation requires spatial colocalisation between CD39^+^ tumour‐reactive T cells and CD73^+^ neighbours rather than resulting from CD39 activity alone. The adenosine generated then acts on A2AR on neighbouring T cells, NK cells and macrophages, including on the CD39‐expressing cell itself, to raise intracellular cAMP, suppress TCR signalling and inhibit cytotoxic granule exocytosis [19, 20]. The immunosuppressive loop is self‐reinforcing: Adenosine further upregulates CD39 and CD73 expression, and hypoxia, which concentrates this process in the ischaemic core of solid tumours, amplifies both enzymes through HIF‐1α [25, 26]. Murine genetic deletion of CD39 specifically from CD8^+^ T cells relieves this suppression and, when combined with anti‐PD‐1 therapy, produces synergistic anti‐tumour effects, providing direct causal evidence that CD39 on TILs is not merely marking exhaustion but enforcing it [25, 26, 27, 28, 29, 30, 31, 32, 33, 34, 35, 36, 37, 38, 39, 40, 41, 42, 43, 44, 45, 46, 47]. Pharmacological blockade of the downstream A2AR recapitulates these results with translational drug‐grade agents [48]. CD39 expression, moreover, is not restricted to tumour‐reactive CD8^+^ TILs: Myeloid cells, B cells, CD4^+^ Th cells, cancer‐associated fibroblasts and most prominently Foxp3^+^ Tregs are known to express CD39 and are also enriched in most solid tumours [42, 49], they all express the ectoenzyme, so that the overall cellular composition of the TME, not CD39^+^CD8^+^ TILs alone, contributes to eATP hydrolysis and adenosine generation. This convergent, multi‐lineage adenosine‐generating activity compounds T‐cell‐intrinsic suppression and raises the therapeutic bar for selective CD39 targeting. Notably, CD39^+^ TILs cluster at the tumour‐immune interface, precisely where bystanders are absent, creating a zone of adenosine‐mediated suppression concentrated where anti‐tumour immunity is most urgently needed. The mechanistic architecture of both faces is illustrated in Figure 1.

**FIGURE 1:**
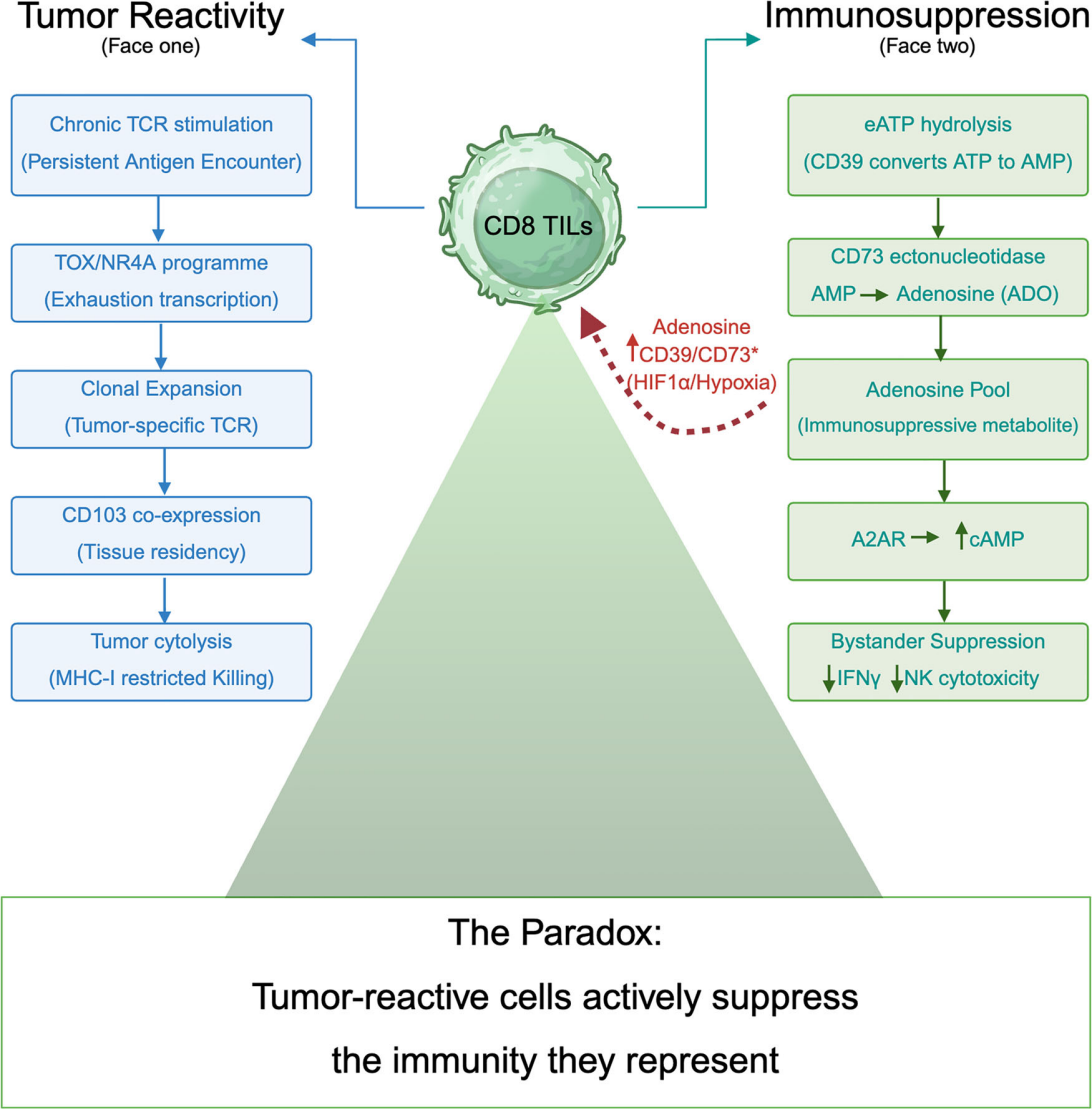
The CD39 paradox on CD8^+^ tumour‐infiltrating lymphocytes. CD8^+^ tumour‐infiltrating lymphocytes (TILs) co‐expressing CD39, CD103, TOX and PD‐1 simultaneously exhibit two functionally opposing identities. Face 1 (left, blue): Chronic TCR stimulation driven by persistent antigen encounter activates the TOX/NR4A transcriptional programme of exhaustion, promoting clonal expansion of tumour‐specific T cells, upregulation of CD103 for tissue residency and ultimately MHC‐I‐restricted tumour cytolysis. Face 2 (right, green): The same cells constitutively hydrolyse extracellular ATP (eATP) via the ectonucleotidase CD39, generating AMP that is further converted to immunosuppressive adenosine by CD73. Direct CD39/CD73 co‐expression on the same tumour‐reactive CD8^+^ T cell has been demonstrated in murine models; in humans, tumour‐reactive CD39^+^CD8^+^ T cells are largely CD73‐negative, and the terminal AMP‐to‐adenosine step instead depends on CD73 expressed by neighbouring tumour cells or other TME populations, making spatial colocalisation—rather than cell‐intrinsic co‐expression—the operative mechanism in human tumours. Accumulation of extracellular adenosine signals through the A2A receptor (A2AR), elevating intracellular cAMP and activating PKA, which suppresses TCR signalling and reduces IFNγ secretion and NK cell cytotoxicity in bystander immune cells. The dashed arrow indicates a positive feedback loop whereby adenosine upregulates CD39 and CD73 expression on CD8^+^ TILs through HIF‐1α stabilisation under hypoxic conditions, thereby amplifying immunosuppression. The paradox: The same tumour‐reactive CD8^+^ TIL population that mediates antigen‐specific cytolysis actively generates an immunosuppressive microenvironment, effectively dampening the broader anti‐tumour immune response it represents.

### 3.3 The Paradox Across Tumour Types: A Cross‐Cancer Analysis

The CD39 paradox does not play out identically across all solid tumours. The balance between the tumour‐reactive signal and the immunosuppressive activity is modulated by the immunological character of the tumour, the degree of hypoxia and the density of competing CD39‐expressing populations. Examining the paradox across tumour types reveals patterns that have not previously been synthesised.

The immunologically hot tumours are defined by high Tumour Mutational Burden (TMB), dense TIL infiltration and responsiveness to ICB, including melanoma, non‐small‐cell lung cancer (NSCLC) and MSI‐high colorectal cancer. Head and neck squamous cell carcinoma (HNSCC), as a whole, is more heterogeneous and is generally regarded as immunologically cold, with the HPV^+^ subset representing the notable hot exception. These tumours show abundant CD39^+^CD103^+^ DP TILs, carry highly expanded tumour‐specific TCR clonotypes and are strongly prognostic for survival [10, 50, 51]. In these tumours, the tumour‐reactive face of CD39 dominates the prognostic signal, and the suppressive face, though present, is partially overridden by the sheer magnitude of the anti‐tumour immune response. CD39 here functions as a reliable tumour‐reactivity biomarker.

In immunologically cold tumours, characterised by sparse TIL infiltration, low TMB, and primary ICB lack of response to ICB, including pancreatic ductal adenocarcinoma (PDAC) and microsatellite‐stable (MSS) colorectal cancer, the picture is more complex. Gorchs et al. [52] demonstrated that CD39^+^CD103^+^CD8^+^ TILs in PDAC are elevated in proliferative rate, co‐express PD‐1 and TIM‐3, and associate with improved survival evidencing that the tumour‐reactive signal survives even in a cold tumour context. Yet the desmoplastic, hypoxic microenvironment of PDAC amplifies HIF‐1α‐driven CD39 and CD73 co‐expression [25], a cell‐intrinsic co‐expression pattern demonstrated in murine models; in human PDAC, CD73 is predominantly expressed by tumour and stromal cells rather than by the CD39^+^ tumour‐reactive T cells themselves, so the suppressive face is more accurately strengthened by HIF‐1α‐driven upregulation of the two ectoenzymes across distinct TME compartments than by co‐expression on the same cell. In MSS colorectal cancer, single‐cell analyses show that CD39^+^ terminally exhausted TILs accumulate progressively across disease stages and are largely absent from the bystander compartment [53, 54], but their functional suppression of the local environment may explain why MSS tumours are ICB‐resistant despite harbouring tumour‐reactive TILs.

Clear cell renal cell carcinoma (ccRCC) is perhaps the most instructive case study for the paradox. Lee et al. [55] recently showed that CD39^+^CD8^+^ TILs in ccRCC are tumour‐antigen specific, satisfying the first face, but simultaneously immunosuppressive through adenosine production and active suppression of bystander T‐cell responses, satisfying the second. This double‐faceted characterisation in a single tumour type and a single cellular population is the clearest empirical validation of the paradox framework proposed in this review. The hypoxic, VHL‐mutant ccRCC microenvironment may represent an extreme case in which HIF‐1α‐driven adenosine production overrides the prognostic benefit of tumour reactivity, explaining the variable outcomes observed in ccRCC patients with high CD39^+^ TIL infiltration [56, 57].

Taken together, these cross‐cancer data support a model in which the tumour‐reactive face of CD39 provides the dominant prognostic signal in immunologically hot or T‐cell‐rich tumours, whereas the suppressive face becomes increasingly dominant and potentially prognostically negative, as the TME becomes colder, more hypoxic or more densely infiltrated by terminally exhausted cells. This hot‐to‐cold framing is likely not the only variable at play; however, as discussed above, CD39 is expressed by multiple TME populations beyond tumour‐reactive CD8^+^ TILs, Tregs, myeloid cells, B cells and CAFs, and CD73, which catalyses the terminal step to adenosine, is itself predominantly supplied by tumour and stromal cells rather than by the T cells themselves. It is therefore plausible that differences in overall TME cellular composition, independent of or in addition to the tumour's position along the hot‐to‐cold spectrum, shape how much CD39 expression on tumour‐reactive CD8^+^ T cells translates into net immunosuppression versus a purely prognostic, tumour‐reactivity signal. A cold tumour with few CD73^+^ or Treg populations might permit CD39^+^ TILs to retain more of their reactive‐face signal, whereas a hot tumour heavily infiltrated by CD73^+^ myeloid cells or CAFs could see the suppressive face amplified despite high TMB and dense infiltration. This model has direct implications for how CD39 should be used as a biomarker and for which patients are most likely to benefit from CD39‐targeted therapy. The spectrum of tumour types along this hot‐to‐cold axis, and the corresponding shift in CD39 face dominance and ICB outcome, is summarised in Figure 2.

**FIGURE 2:**
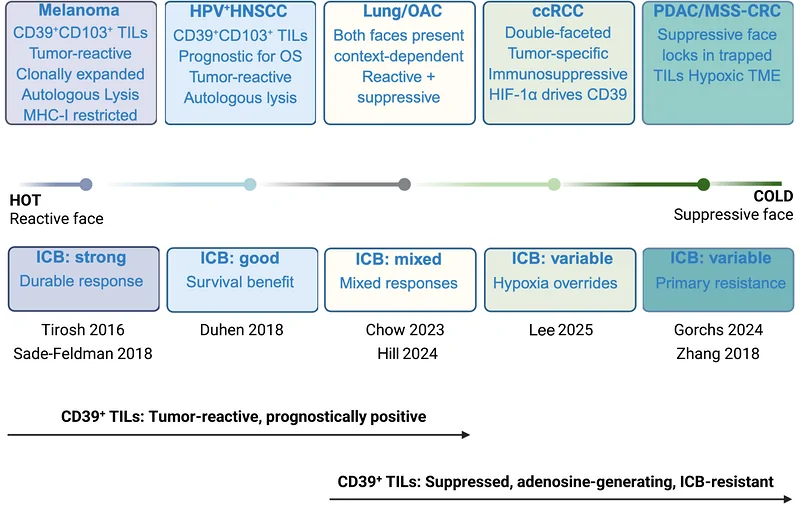
The CD39 paradox across solid tumour types: a hot‐to‐cold spectrum. Tumour types are arrayed from immunologically hot (left, blue) to cold (right, green). Above the axis: the functional identity of CD39^+^ TILs reactive‐dominant, balanced or suppression‐dominant. Below: ICB outcome. In hot tumours (melanoma, HPV^+^ HNSCC), CD39^+^CD103^+^ TILs are clonally expanded and prognostically positive. In ccRCC, TILs are tumour‐specific yet immunosuppressive the purest expression of the paradox. In cold tumours (PDAC, MSS‐CRC), HIF‐1α‐driven CD39 and CD73 upregulation locks in the suppressive face, explaining primary ICB resistance. ccRCC, clear cell renal cell carcinoma; HNSCC, head and neck squamous cell carcinoma; HPV, human papillomavirus; ICB, immune checkpoint blockade; MSS‐CRC, microsatellite‐stable colorectal cancer; PDAC, pancreatic ductal adenocarcinoma.

## 4 CD39 and the Architecture of T‐Cell Exhaustion

T‐cell exhaustion spans a continuum from proliferative, ICB‐responsive progenitor‐exhausted cells (T*pex*, TCF‐1^+^) to epigenetically fixed terminally exhausted cells (T*tex*) expressing high co‐inhibitory receptor levels and largely refractory to reinvigoration [58, 59, 60, 61, 62, 63]. CD39 maps predominantly onto the T*tex* pole, confirming tumour‐antigen encounter while marking the cells least able to be directly rescued by PD‐1 blockade [29, 30, 31]. This positioning is clinically decisive: A T*pex*‐rich tumour is one where the reactive face of CD39 can still be reinvigorated by ICB; a T*tex*‐dominated tumour is one where the suppressive face has locked in, and CD39's epigenetically reinforced expression [64] means that enzymatic blockade alone, without epigenetic reprogramming, will not restore full effector competence.

## 5 Tissue‐Resident Memory T and NK Cells: A Parallel Local Immune Compartment

The framework developed above centres on circulating‐derived, tumour‐antigen‐experienced CD8^+^ TILs, but it does not account for a second, largely non‐recirculating compartment that dominates local immunity in many peripheral organs: tissue‐resident memory T and NK cells (TRM). In barrier and interface tissues, most notably the gut, skin, liver and lung, TRM populations establish long‐lived, organ‐adapted immune surveillance that is largely independent of the recirculating T‐cell pool [65, 66]. This tissue‐resident compartment accumulates and is progressively maintained over decades of cumulative antigenic exposure, becoming a dominant, stable component of the local T‐cell pool in barrier organs by adulthood [67]. This is particularly relevant to solid tumours arising within or adjacent to such interface organs, where the pre‐existing local TRM repertoire may constitute a substantial component of the local baseline T‐cell pool, even though anti‐tumour immunity is thought to be driven predominantly by T cells primed in the tumour‐draining lymph nodes and recruited via the circulation, rather than by the pre‐existing TRM repertoire itself.

TRM T and NK cells share a substantial phenotypic overlap with tumour‐reactive CD8^+^ TILs, including PD‐1, CD49a and CD69 expression and, directly relevant to this review, CD39 itself, which is expressed by a subset of CD69^+^CD103^+^ TRM, particularly in barrier and interface tissues, across multiple non‐lymphoid tissues in both mice and humans [68]. Notably, unlike the dysfunctional CD39^+^ populations that define chronic‐infection and tumour exhaustion, CD39^+^ TRM remain fully functional upon ex vivo antigen restimulation, underscoring that CD39 expression per se does not equate to exhaustion outside the tumour‐antigen‐chronic‐stimulation context central to this review. This convergence is functionally significant: In several organs, TRM cells represent the major T‐cell subset responding to immune checkpoint inhibitor (ICI) therapy, independent of classical circulating memory populations. However, the same work shows that virus‐specific bystander TRM in tumours can express CD39 at levels comparable to tumour‐reactive CD8^+^ T cells, complicating the assumption, central to Face 1 of the paradox, that CD39 reliably discriminates tumour‐reactive from bystander cells in TRM‐rich tissues. Taken together, this raises the specific hypothesis that, in interface organs, the CD39^+^ TRM‐like fraction may represent a principal, but not unambiguously tumour‐specific, component of the T‐cell pool mediating ICI response, situating tissue residency as a third axis, alongside the hot‐to‐cold spectrum discussed in Section 3.3, along which the CD39 paradox plays out, while also flagging a boundary condition the model must account for.

## 6 Clinical and Prognostic Significance

### 6.1 Reading the Paradox Prognostically

The dual identity of CD39^+^CD8^+^ TILs creates an interpretive challenge for prognostic frameworks. The immune contexture, the density, type and localisation of immune cells in the tumour is among the most robust prognostic variables in human cancer, outperforming conventional Tumour, Node, Metastasis (TNM) staging in colorectal cancer and correlating with survival across multiple malignancies [69, 70, 71, 72]. Within this framework, CD39 adds a layer of functional specificity: It separates the tumour‐reactive signal from the bystander noise. In HNSCC, PDAC, OAC (oesophageal adenocarcinoma) and lung cancer, higher frequencies of CD39^+^CD103^+^ DP TILs consistently correlate with improved overall survival [10, 51, 52, 73], reflecting the prognostic value of having a quantifiable tumour‐reactive T‐cell response. This benefit is recapitulated across the tissue‐residency literature [74, 75, 76, 77, 78, 79].

### 6.2 Predicting Response to ICB

If CD39 marks the tumour‐reactive CD8^+^ TIL compartment, it should predict ICB response, and the evidence largely supports this. Chow et al. [11] showed in lung cancer that CD39‐high TILs, identified by CITE‐seq, harboured the highest TCR clonal expansion and the most pronounced transcriptomic signatures of tumour reactivity, and that their frequency correlated with clinical response to anti‐PD‐1 therapy. This makes intuitive sense: ICB reinvigorates the progenitor‐exhausted (T*pex*) fraction [38, 40, 58], and the CD39^+^ terminally exhausted cells are the progeny that the reinvigorated T*pex* cells would generate after antigen re‐encounter. The presence of many CD39^+^ TILs therefore signals that there is a meaningful tumour‐reactive pool capable of responding to checkpoint reinvigoration, even if those specific CD39^+^ cells are themselves beyond reinvigoration. This interpretation reconciles the apparent paradox of terminal exhaustion as a positive biomarker [39, 80, 81].

## 7 Therapeutic Targeting: Exploiting the Paradox

### 7.1 Blocking the Suppressive Face: Anti‐CD39 and Downstream Agents

The paradox generates a clear therapeutic logic. If CD39^+^CD8^+^ TILs are tumour‐reactive but self‐suppressing, then blocking CD39 enzymatic activity without depleting or impairing the tumour‐reactive cells that express, it should simultaneously restore eATP‐mediated pro‐inflammatory signalling and reduce adenosine‐mediated suppression, converting these cells from bystander suppressors to active effectors. This logic is supported by preclinical data: Pharmacological blockade or genetic deletion of CD39 in murine tumour models restores T‐cell effector function, increases intratumoural eATP, activates the NLRP3 inflammasome–IL‐18 axis, and synergises with anti‐PD‐1 checkpoint blockade [45, 46].

The choice of therapeutic modality is not neutral to this logic, however, and deserves explicit consideration given how widely CD39 is expressed across the TME. CD39‐depleting antibodies eliminate the target cell along with its enzymatic activity: This is an attractive strategy when the goal is to remove immunosuppressive CD39‐high Tregs or myeloid populations but applied to tumour‐reactive CD8^+^ TILs it would remove the very effector cells the paradox model seeks to rescue, working against rather than with the therapeutic logic above. Antibodies that block CD39 enzymatic activity without depleting the expressing cell, by contrast, are better aligned with rescuing tumour‐reactive CD8^+^ T cells specifically, as they leave the cell, and its antigen‐experienced, tissue‐resident identity, intact while shutting down eATP catabolism. Small‐molecule A2AR antagonists act further downstream and are agnostic to which cell produced the adenosine, making them a rational choice precisely because CD39 (and CD73) expression is distributed across so many TME lineages: Rather than trying to selectively target the T‐cell‐intrinsic source, an A2AR antagonist neutralises the convergent output of the whole multi‐lineage adenosine‐generating network at once, at the cost of losing that cellular selectivity. The optimal strategy may therefore depend on tumour composition: Enzymatic‐blocking or A2AR‐antagonist approaches would be preferable when tumour‐reactive CD39^+^CD8^+^ TILs are abundant and worth preserving, whereas depleting antibodies may still have a role where CD39‐high Tregs or suppressive myeloid cells dominate the CD39^+^ compartment.

Multiple clinical‐stage agents target this pathway. Anti‐CD39 monoclonal antibodies (Phase I/II; including TTX‐030 and SRF617) block enzymatic activity or deplete CD39‐high Treg populations. Anti‐CD73 antibodies (MEDI9447/oleclumab, BMS‐986179) and small‐molecule A2AR antagonists (CPI‐444, AZD4635) are being evaluated in combination with PD‐1/PD‐L1 blockade, with early signals of immune activation and manageable toxicity [82, 83, 84].

### 7.2 Exploiting the Reactive Face: CD39 in Adoptive Cell Therapy (ACT)

If CD39 marks tumour‐reactive TILs, it can be used not just as a biomarker but as a sorting handle for the manufacture of ACT products. Fluorescence‐activated cell sorting using the CD39^+^CD103^+^ DP gate from surgical tumour specimens enriches for tumour‐reactive clonotypes [10, 85] and could substantially improve the therapeutic potency of TIL‐ACT products relative to unfractionated bulk TIL expansions, which have demonstrated durable responses in melanoma and HPV‐associated cancers [86]. Proof‐of‐concept studies in HNSCC and colorectal cancer confirm that DP‐sorted TILs contain higher proportions of tumour‐reactive clones and mediate more effective autologous tumour killing than unsorted populations [10]. This translational rationale is now being tested clinically: An ongoing first‐in‐human Phase I trial is evaluating a CD39^+^CD103^+^ DP‐sorted TIL product as ACT in patients with metastatic solid tumours [87].

The major challenge for CD39‐enriched ACT is the epigenetically locked exhausted state of sorted DP TILs. Their re‐expansion ex vivo is technically feasible but risks deepening exhaustion through further TCR stimulation and metabolic stress [88, 89, 90]. Optimised culture strategies as reduced‐dose IL‐2, IL‐21 supplementation, anti‐PD‐1 co‐blockade during expansion and careful mitochondrial preservation [91], have been shown to partially maintain the tumour‐specific TCR content and effector capacity of CD39^+^PD‐1^+^ TIL fractions during expansion [92].

Optimising expansion protocols for CD39‐enriched TIL fractions remains an active area of investigation; whether CD39 enzymatic blockade during ex vivo culture could itself mitigate autocrine suppression and improve product quality is an open and clinically relevant question.

## 8 Conclusions and Future Perspectives

The biology of CD39 on CD8^+^ TILs encapsulates a paradox that has practical consequences for how tumour‐reactive T cells are identified, interpreted and targeted. The molecular features that make CD39 a reliable marker of genuine tumour‐antigen encounter, chronic TCR stimulation, TOX‐driven transcriptional exhaustion and clonal expansion within the tumour are the same features that couple its expression to active adenosine generation, creating a biochemical loop in which the most immunologically relevant cells enforce their own suppression and that of their neighbours [93, 94]. This duality explains why CD39^+^ TIL density can simultaneously predict ICB responsiveness [95, 96, 97, 98, 99, 100] and correlate with functional impairment, and why the prognostic value of CD39 is context‐dependent rather than absolute [101, 102].

Two mechanistic questions remain unresolved. Whether CD39 induction is upstream or downstream of the TOX/NR4A exhaustion programme [9, 32, 64, 98, 103, 104], or whether the relationship is bidirectional, has not been established in primary human TILs; CRISPR‐mediated ENTPD1 deletion is beginning to address causality [46, 105], but population‐level and single‐cell perturbation data in human tissue remain sparse. The spatial organisation of the CD39 paradox remains equally poorly resolved: Current evidence derives predominantly from dissociated tumour preparations, and whether the adenosine‐generating relay formed by CD39^+^CD8^+^ TILs, CD39^+^ Tregs [106, 107, 108] and CD73^+^ stromal cells operates as a spatially coordinated suppressive unit, rather than as independently distributed components, has not been directly demonstrated [109, 110, 111, 112, 113]. Spatially resolved transcriptomics in prospective ICB trial biopsies will be necessary to test this [114, 115].

The therapeutic implication is the enzymatic blockade of CD39 that targets the suppressive function of tumour‐reactive TILs without eliminating them, a property that distinguishes it from both checkpoint blockade and Treg‐depleting strategies. Realising this potential will require patient stratification based on the relative dominance of the two faces, tools that combine CD39 expression, spatial context and exhaustion depth, and manufacturing protocols for CD39‐enriched ACT that preserve effector competence through epigenetic co‐intervention [116]. The paradox, properly understood, is not an obstacle to therapeutic targeting of CD39 but the rationale for it.

## Author Contributions

Manolo Sambucci conceived and organized the review and wrote the manuscript. Daniela Fenoglio, Giovanna Borsellino, Luca Battistini, and Gilberto Filaci critically reviewed the manuscript. Francesca Ferrera, Laura Camporesi, and Andrea Lagazio reviewed the figures and the bibliography. All authors read and approved the final manuscript.

## Funding

The authors have nothing to report.

## Conflicts of Interest

L.B. declares personal compensation for consultancy, research grants and support for attendance at conferences from Bristol Myers Squibb, Merck, Novartis, Roche Sanofi, Janssen, Horizon, Amgen, Argenx and GlaxoSmithKline. The other authors declare no conflicts of interest.

## Data Availability

Data sharing is not applicable to this article as no datasets were generated or analysed during the current study.
